# Notes and News

**Published:** 1898-08-20

**Authors:** 


### Notes and News.

(Collected and Communicated.)

A new wing has been added to the Moreton Hampstead Convalescent Home. The building now contains accommodation for 22 inmates.

Rural Guardians have received an intimation that the Conscience Clause in the Vaccination Bill will come into effect early in the coming year.

Colonel Roosevelt and the first detachment of American volunteer cavalry have been landed at New York, and are to be kept in close quarantine for four or five days.

A new infirmary has been opened at Hebburn. The building was originally known as Hebburn Hall, and has now been converted into an infirmary to contain 28 beds. One large ward contains 20 beds for male patients. The smaller ward is for women. An operating room, dispensary, and the usual accommodation for the staff has been provided. Mr. Care-Ellison, to whose family Hebburn Hall previously belonged, performed the opening ceremony.

Clinical instruction is given at the Royal Ear Hospital, Frith Street, by the members of the staff. Students are admitted to the practice and teaching on the payment of one guinea per six weeks' course, this allowing of two attendances in each week. The next course will commence on October 3rd. Notice of this and of subsequent courses will be found in the medical journals. The teaching is purely practical. For further information apply to the hon. secretary of the Medical Board.

We are accustomed on this side of the world to associate direct advertising with very low class practice. In New Zealand things are managed somewhat differently. The following little announcement is extracted by the *Australian Medical Gazette* from the *Hobart Mercury* for March 7th, 1898: "Dr. A. Crosbie Dixey, Member Royal College Physicians, Edinburgh, wishes to announce he has started a consulting practice in New Norfolk. Specialities—Typhoid and other fevers, and diseases of women and children. 'Woodbridge,' New Norfolk." Consulting practice, indeed! And what a choice of specialities! Both science and ethics have here gone sadly astray.

The health of the British troops at Atbara is reported good. A few cases of sunstroke have occurred.

The great heat in London, which has been unprecedented for a long time past, has been the cause of a number of fatal cases of sunstroke.

The Prince of Wales is doing well, and it is expected that he will go for a cruise almost immediately.

It would seem that Pink Pills for Pale People has proved a very attractive advertisement, since a rival alliteration has found its way into Australian newspaper advertisements. Sentiment is entirely removed from the last advertisement, which temptingly describes the remedy referred to as "Bile Beans for Biliousness."

Of course it is too early for any very great accession in the number of candidates for the Royal Army Medical Corps to manifest itself since the warrant instituting the corps has been issued. Still, the number of candidates and vacancies at the recent examination for commissions was so nearly even that, as soon as a fresh number of students are ready, a much more appreciable enthusiasm for the service is expected.

A new wing has been added to Warminster Cottage Hospital through the generosity of Miss Smith, of Warminster. The donor is devoting £1,000 to the building and giving a further £4,000 to create a maintenance fund. The present building contains 14 beds, and the addition will provide not only for such extra beds as are required, but for offices and rooms which will render the hospital far more complete and commodious.

The Queen has invested Dr. Andrew Milroy Fleming, medical director of the British South Africa Company and principal medical officer of the British South African Police, with a Commandership of the Order of St. Michael and St. George. This honour has been awarded him in recognition of his services in connection with operations in Mashonaland last year. Surgeon-Lieutenant-Colonel F. MacCartre, M.B., of the Indian Medical Service Corps, has received the Companionship of the Order of the Indian Empire also from the hands of the Queen.

Another case of the fatal confusion which occurs between drunk and dying persons admitted into hospitals has occurred in the case of James Fisher, a carman, who fell from his van at Islington, and was taken to Guy's Hospital, and was attended by the house surgeon. The condition of the man was diagnosed as intoxication, and the police-constable in whose care he had been brought to the hospital eventually removed him to the police-station, where he was locked up. On the divisional surgeon visiting him, he had him at once removed to the Rotherhithe Infirmary, where he died from the results of a fracture of the skull. The coroner, at the inquest, added to the verdict of Accidental death the remark that the house surgeon at Guy's had not shown adequate judgment in his diagnosis and the recommendation, which has been so frequently meted out of late to the hospitals, that they should retain doubtful cases of intoxication until any mistake in the diagnosis was impossible.

